# Parastomal Varices with Recurrent Bleeding in the Absence of Liver Cirrhosis

**DOI:** 10.1155/2020/2653848

**Published:** 2020-09-14

**Authors:** Jonathan Kopel, Rebeccah Baucom, Samuel Campbell, Gregory L. Brower

**Affiliations:** ^1^Texas Tech University Health Sciences Center School of Medicine, Lubbock, TX, USA; ^2^Department of Surgery, Texas Tech University Health Sciences Center, Lubbock, TX, USA; ^3^Department of Surgery Division of Vascular Surgery, Texas Tech University Health Sciences Center, Lubbock, TX, USA; ^4^Department of Medical Education, Texas Tech University Health Sciences Center, Lubbock, TX, USA

## Abstract

Gastrointestinal (GI) bleeding is a common problem in patients with portal hypertension. One of the most common causes of GI bleeding are varices (e.g., esophageal varices). In some instances, varices can develop between an intestinal stoma and the abdominal wall vasculature, known as parastomal varices. Specifically, parastomal varices are common in patients with a preexisting stoma and concurrent chronic portal hypertension. These patients often present with recurrent bleeding and may require regular transfusions. Herein, we report on a patient with parastomal varices and portal hypertension without hepatic cirrhosis. Given the high morbidity and mortality associated with surgical interventions, most clinical guidelines encourage observation and medical management of bleeding from parastomal varices. Among the nonsurgical interventions, manual compression and local maneuvers often successfully stop the bleeding. However, subsequent rebleeding from parastomal varices can remain a problem requiring additional treatment. Further research is needed to investigate appropriate medical or surgical alternatives for managing parastomal varices bleeding.

## 1. Introduction

Varices are a common cause of gastrointestinal (GI) bleeding in patients with portal hypertension [1]. In most cases, varices will develop in three primary locations: (1) the left gastric and esophageal veins, (2) the superior, middle, and inferior rectal veins, and (3) the paraumbical veins [2]. However, varices can also develop between an intestinal stoma and the abdominal wall vasculature, with the resulting parastomal varices being particularly common among portal hypertension patients [2]. Peristomal varices occur in 27%–50% of patients with portal hypertension and a preexisting stoma [3]. In most cases, parastomal varices appear as circumferential blue or purple subcutaneous rings extending from the mucocutaneous junction of the subcutaneous stomal skin and present with external bleeding, which may be life-threatening [3]. In this case report, we discuss a patient with parastomal varices and portal hypertension without known liver cirrhosis.

## 2. Case Report

A 56-year-old man presented to the emergency department with bleeding (>700 cc) around his colostomy site persisting for two days. The patient denied any pain with the bleeding, fever, chills, sweats, weakness, abdominal pain, nausea, vomiting, or diarrhea. The patient denied any history of smoking, alcohol, or any other substance abuse. The patient's surgical history included a remote colectomy with permanent colostomy for stage IV colon cancer and a subsequent colostomy revision and hernia repair (8 and 6 years, respectively, before this episode), after experiencing a massive bleed from a parastomal varices. The patient has a medical history of hypertension, hyperlipidemia, diabetes, hypothyroidism, colon cancer, obstructive sleep apnea, and obesity.

The patient's current medications upon presentation in the emergency department included pioglitazone, ipratropium bromide, insulin glargine, montelukast, levocetirizine, atorvastatin, azelastine, bisoprolol, levothyroxine, lisinopril, metformin, triamcinolone, and aspirin. The patient's blood pressure was 128/84 mmHg. The patient's white blood cell count was 3,970 cells/*µ*L, platelet count was 119,000/*µ*L, and red blood cell count was low at 4.080 million cells/uL. His hemoglobin and hematocrit levels were 13.7 g/dL and 39.2%, respectively. His sodium was below normal, and he was hypochloremic (135 mmol/L and 95 mmol/L, respectively), glucose was elevated at 313 mg/dL, and creatine was normal at 0.8 mg/dL. His liver function was normal, aside from slightly elevated alanine transaminase of 64 international units/L.

Given the recurrent bleeding over several days, surgery was consulted to evaluate the bleeding. No further bleeding was elicited, and the patient was discharged. However, the patient returned to the emergency department the next day reporting 50 cc of dark red blood while emptying his colostomy bag. Vascular surgery and gastroenterology were consulted. A right upper quadrant ultrasound demonstrated a gallstone, no pericholecystic fluid or wall thickening, and common bile duct diameter of 6 mm without intrahepatic biliary ductal dilatation. The liver measured 20 cm in length with the portal vein slightly dilated at 1.1 cm. Furthermore, the portal vein showed hepatopedal blood flow with a velocity of 14.6 cm/s. The liver echotexture was also hyperechogenic suggesting mild hepatic steatosis. Visualization of the pancreas was normal.

**Figure 1:**
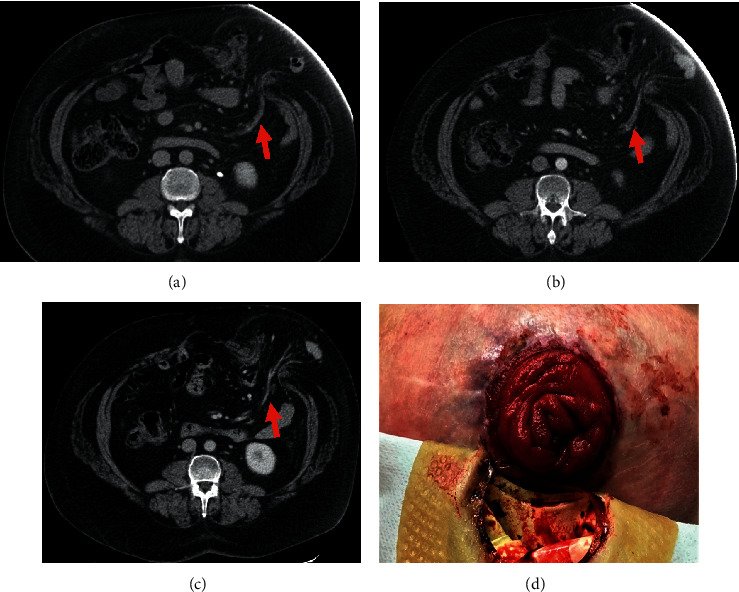
(a–c) Abdominal CT scans demonstrating dilated parastomal varices (red arrows). (d) Image depicting parastomal varices and hernia.

The colonoscopy performed through the stoma was without abnormality. EGD showed a normal esophagus without any esophageal varices and a normal duodenum; however, mild portal hypertensive gastropathy was found diffusely in the entire stomach. A CT scan of the abdomen showed the main portal vein to be slightly dilated measuring 14 mm. The superior mesenteric vein was also dilated measuring 15 mm. Mesenteric veins extending into the parastomal hernia were also dilated. As shown in Figure 1, the CT scan demonstrated a parastomal hernia with accompanying dilated parastomal varices. Given the lack of further bleeding, the patient was released and given instructions on how to reduce the risk of further bleeding from the parastomal varices. The patient subsequently had another episode of massive bleeding (>700 cc) occurring two years after the incident described herein, which resolved sponstaneously after the patient aggressively debrided the vascular erosion and applied manual compression. No rebleeding has occurred in the year since the last incident.

## 3. Discussion

Obstruction of the portal venous blood flow is a well-known mechanism leading to portal hypertension in between high- and low-pressure systemic venous systems [2,3]. Patients with portal hypertension who have a colostomy or ileostomy may develop collateral venous channels to the anterior abdominal wall forming parastomal varices [2]. In most instances, parastomal varices branch from mesenteric branches off the superior mesenteric vein [7]. In this case report, the patient had a history of stage IV colon cancer requiring the formation of a permanent colostomy. The patient subsequently developed parastomal varices with a parastomal hernia. As shown in Table 1, most patients with parastomal varices have a history of ulcerative colitis, carcinoma of rectum, carcinoma of urinary tract, liver cirrhosis, primary sclerosing cholangitis, or hepatitis. However, the patient in this case presented with a history of colon cancer with portal hypertension without known liver cirrhosis.

The bleeding from parastomal varices can be a slow venous ooze without a focal source or a pressurized spurting bleed [7]. The bleeding resulting from portal hypertension can be massive and cannot always be manually compressed [7]. When bleeding is focal, manual compression with a single-digit at the bleeding site or outside the stoma may provide temporary control [7]. The parastomal varices in this patient showed focal bleeding that was controlled by applying pressure using a cotton swab. Recent case reports have shown the use of percutaneous *N*-butyl-2-cyanoacrylate glue injection effectively stopped bleeding from parastomal varices [8]. However, injection of *N*-butyl-2-cyanoacrylate glue is not approved in the United States [1]. Other case reports have used a combination of sodium tetradecyl sulfate, ethanol, and saline to reduce the number of blood transfusions [1]. Polidocanol, 5% phenol, and nonselective beta-blockers have also shown some beneficial reduction in parastomal varices bleeding [1].

Despite being common in patients with a colostomy, many patients and physicians are unaware of parastomal varices and the frequency of bleeding [2,8]. Physicians are encouraged to remove any bandages or stoma appliance to examine the surrounding skin for a purplish hue around the stomal area, which suggests presence of parastomal varices [8]. However, only 25% of patients exhibit the purplish hue; physicians are encouraged to use Doppler ultrasound, contrast-enhanced CT, and portal vein venography to confirm the diagnosis for many patients with recurrent bleeding around the stoma site [2]. Furthermore, direct suture repair or stoma relocation is not recommended due to the high rate of recurrence (60%) [4].

Although no standard treatment for bleeding from parastomal varices has been established, the current report suggests that conservative management of parastomal varices using manual compression, with or without focal debridement, should be given consideration. However, because this approach frequently fails to effectively control the bleeding, several other treatment options have been used to treat parastomal varices, including nonoperative treatment, local operative treatment, surgical portosystemic shunts, embolization (transhepatic or transjugular), transjugular intrahepatic portosystemic shunt, and liver transplant [2]. In recent years, alternative surgical techniques, such as extraperitoneal tunneling, stapled ostomy creation, stoma-fascia fixation, and prophylactic mesh reinforcement, were used to prevent the development of parastomal varices [5]. A meta-analysis including 1,071 patients who had a colostomy created using either the extraperitoneal versus intraperitoneal techniques found a lower rate of parastomal hernias in the extraperitoneal group [9].

If bleeding remains persistent or increases in severity, reducing portal hypertension using a transjugular intrahepatic portosystemic shunt (TIPS) effectively prevents most rebleeding events from parastomal varices [8]. Specifically, only 20% of patients with parastomal varices with a history of rebleeding continued to have bleeding after the TIPS procedure [2]. However, a TIPS procedure is contraindicated for patients with primary or metastatic liver cancer or for those with advanced liver cirrhosis. Currently, over 75% of patients with parastomal varices and bleeding are treated medically using nonselective beta-blockers. Despite surgical advances in treatment, patients with parastomal varices have a high risk of morbidity (13%), mortality (6.3%), and recurrence (11% after 3 years) [4]. Given the significant number of affected patients, further research is needed to investigate appropriate medical or surgical approaches for managing parastomal varices bleeding.

## Figures and Tables

**Table 1:** Summary of parastomal varices risk factors, presentation, and treatment [4–6].

Patient risk factors	Surgical risk factors	Appearance	Clinical presentation	Treatment
(i) Obesity (ii) Malnutrition (iii) Advanced age (iv) Smoking (v) Collagen abnormalities (vi) Corticosteroid use (vii) Postoperative wound sepsis (viii) Ascites (ix) Abdominal distention (x) Chronic constipation (xi) Obstructive uropathy (xii) Chronic obstructive lung disease	(i) Inappropriate stoma site selection (ii) Oversized fascial trephine (iii) Excessive splitting and stretching of abdominal rectus muscle (iv) Epigastric nerve denervation (v) Emergency stoma creation	Circumferential blue or purple subcutaneous rings extending from the mucocutaneous junction of the subcutaneous stomal skin	(i) Peristomal esthetic complaints (ii) Discomfort (iii) Pain (iv) Bowel obstruction (v) Incarceration	(i) Prophylactic synthetic mesh placement (ii) *N*-Butyl-2-cyanoacrylate glue injection (iii) Single-digit compression (iv) Sodium tetradecyl sulfate (v) Polidocanol (vi) 5% phenol (vii) Nonselective beta-blockers (viii) Liver transplant (ix) Embolization (x) Transjugular intrahepatic portosystemic shunt

## Data Availability

No additional experimental data were used to support the conclusions of this study.
